# Sexual Success after Stress? Imidacloprid-Induced Hormesis in Males of the Neotropical Stink Bug *Euschistus heros*

**DOI:** 10.1371/journal.pone.0156616

**Published:** 2016-06-10

**Authors:** Khalid Haddi, Marcos V. Mendes, Marcelo S. Barcellos, José Lino-Neto, Hemerson L. Freitas, Raul Narciso C. Guedes, Eugênio E. Oliveira

**Affiliations:** 1 Departamento de Entomologia, Universidade Federal de Viçosa, Viçosa, MG, 36570–900, Brasil; 2 Science without Border Associate Researcher, Programa de Pós-Graduação em Entomologia, Universidade Federal de Viçosa, Viçosa, MG, 36570–000, Brasil; 3 Departamento de Biologia Geral, Universidade Federal de Viçosa, Viçosa, MG, 36570–900, Brasil; French National Institute for Agricultural Research (INRA), FRANCE

## Abstract

Environmental stress in newly-emerged adult insects can have dramatic consequences on their life traits (e.g., dispersion, survival and reproduction) as adults. For instance, insects sublethally exposed to environmental stressors (e.g., insecticides) can gain fitness benefits as a result of hormesis (i.e., benefits of low doses of compounds that would be toxic at higher doses). Here, we experimentally tested whether sublethal exposure to the insecticide imidacloprid would hormetically affect the sexual fitness of newly-emerged adults of the Neotropical brown stink bug *Euschistus heros* (Hemiptera: Heteroptera: Pentatomidae), which is the most abundant and prevalent insect pest in Neotropical soybean fields. We evaluated the sexual fitness of four couple combinations: unexposed couples, exposed females, exposed males, and exposed couples. Sublethal exposure to dry residues (i.e., contact) of imidacloprid (at 1% of recommended field rate) did not affect insect survival, but led to higher mating frequencies when at least one member of the couple was exposed. However, the average mating duration was shortened when only females were exposed to imidacloprid. Moreover, exposed males showed higher locomotory (walking) activity, lower respiration rates and induced higher fecundity rates when mated to unexposed females. Although the reproductive tracts of exposed males did not differ morphometrically from unexposed males, their accessory glands exhibited positive reactions for acidic and basic contents. Our findings suggest that males of the Neotropical brown stink bug hormetically increase their sexual fitness when cued by impending insecticidal stress in early adulthood.

## Introduction

Events occurring in the early adulthood of insects can have substantial effects on their life traits (e.g., dispersion, survival and reproduction) as adults. Several studies on insects have shown that stress early in life can increase longevity, stress resistance (e.g., insecticide tolerance, temperature challenges, fungal infection, and food and oxygen deprivation) and sexual success [1–7].

In agricultural ecosystems all over the globe, most organisms are repeatedly exposed to insecticide-mediated stresses from multiple sources throughout their lifetimes, potentially affecting different aspects of organismal performance. Such inevitable exposure derives from the fact that one of the fundamental tenets of pest management is the deliberate use of insecticides aimed at reducing pest populations as much as possible, thereby minimizing losses in yield [3,8]. However, once these insecticide molecules are poured on such ecosystems, they are likely to act on other non-targeted organisms or their interactions with the target pests may occur at lower doses, which may not lead to direct mortality but instead may result in sublethal consequences that potentially interfere with their survival and/or reproduction [3,4,9–14]. When these sublethal exposures to insecticides lead to stimulatory responses in exposed insects, it is referred as insecticide-induced hormesis, which has been identified in a number of arthropod species by influencing their reproductive output [9,15–19].

Beneficial effects of hormesis are often, but not always, greater in males than in females [20]. Curiously, the contribution of males to insecticide-induced hormetic responses on the insect sexual success has been largely ignored. Male insects sublethally exposed to insecticides may alter their abilities of enticing or coercing the females for multiple matings and, therefore, increase the probability of siring offspring [21–25]. Furthermore, by increasing locomotory activities, males may increase their sexual success due to increases in the rate of encounters between mating partners [26]. However, increased mobility may also result in additional encounters with predators [27], which can negatively affect male sexual success. Moreover, higher mobility may increase the chances that pests will evade insecticide-contaminated areas [28,29], but this ability may also lead to increased respiratory activities (i.e., O_2_ uptake/CO_2_ release), which, in such environments, may increase the chance of auto-intoxication [30] and impairment of oxidative phosphorylation processes [31–33].

Among insecticides registered for agricultural applications, the neonicotinoids (with imidacloprid being the most notable example) is a fast-growing class of insecticides because these molecules have shown low levels of cross resistance to conventional long established insecticide classes (e.g., organochlorides, organophosphates, carbamates, pyrethroids) [34]. These molecules act selectively on the insect central nervous system by disrupting the functions of the different subtypes of nicotinic receptors nAChRs [34–38]. In Brazil, for instance, neonicotinoids have become the most common insecticides used to control several sap-sucking insects, such as the Neotropical brown stink bug *Euschistus heros* (F.) (Hemiptera: Heteroptera: Pentatomidae) [39,40], which is the most abundant and prevalent insect pest in Neotropical soybean fields [41–43]. In a recent investigation, Santos et al. [44] demonstrated that females of *E*. *heros* sublethally exposed to imidacloprid had enhanced reproductive output, which suggests that there is a potential link between sublethal exposure to imidacloprid and recent outbreaks of *E*. *heros* observed in Brazilian soybean fields.

In the present investigation, we assessed the sexual success of *E*. *heros* males by evaluating the reproductive output of couples where either one or both members survived imidacloprid-induced stress in early adulthood. By taking morphometric measurements of male reproductive tracts and of physiological (e.g., CO_2_ release) and behavioral (e.g., walking) activities of males after being sublethally exposed to imidacloprid, we are contributing to a better understanding of the underlying mechanisms involved in differences in the sexual success of both sexes in response to insecticidal stress.

## Material and Methods

### Insect collection and rearing

A colony of *E*. *heros* was established from eggs obtained from the Semiochemical Laboratory of the EMBRAPA Natural Resources and Biotechnology (Brasília, DF, Brazil). The colony was multiplied and reared under controlled conditions (27 ± 2°C, 60 ± 20% relative humidity, with an L:D photoperiod of 14:10 h). To prevent diapause, artificial lighting was maintained between 08:00 and 22:00 h. All of the developmental stages of *E*. *heros* were mass-reared in plastic boxes following methods previously described elsewhere [45–47]. Field-collected individuals from soybean farms at the Tangará da Serra region (State of Mato Grosso, Brazil) and from the experimental soybean fields at the Federal University of Viçosa (UFV; Viçosa, State of Minas Gerais State, Brazil) were routinely introduced into the laboratory colony to increase the genetic variability of insects used in the experiments. The field-collections were carried out on private lands (with the permission of their owners) and at the UFV experimental fields by personnel of UFV, and no specific permissions were required for these locations/activities as it did not involve endangered or protected species. All applicable international, national, and institutional guidelines for the care and use of the insects were considered in the present investigation

The species studied is an herbivorous pentatomid species from a colony maintained in laboratory, where the experiments were performed and no specific permission was required. The field-collections were carried out on private lands (with the permission of their owners) and at the Federal University of Viçosa (UFV) experimental fields by personnel of UFV, and no specific permissions were required for these locations/activities as it did not involve endangered or protected species. All applicable international, national, and institutional guidelines for the care and use of the insects were considered in the present investigation.

### Exposure to sublethal doses of imidacloprid

Newly emerged (< 24 h old) groups of 50 adult males and 50 adult females of *E*. *heros* were exposed separately for 48 h to dry imidacloprid residues (at 0.042 μg a.i./cm^2^, the equivalent of 1% of the field label rate) or to deionized water (control). Preliminary bioassays indicated that this concentration was the highest one with no observable effects on newly emerged adult survival of *E*. *heros* [44]. After the 48 h exposure time, females and males were kept in separate plastic containers for 10 days to reach the sexual maturity. The tops of the containers were sealed with a piece of organza veil and a rubber band to prevent insects from escaping. The bottom was covered with paper towels to absorb moisture. All of the insects were fed *ad libitum* with a mixture of the fresh pods of green beans (*Phaseolus vulgaris* L.), dry soybean seeds (*Glycine max* L.), raw shelled peanuts (*Arachis hypogaea* L.) and sunflower seeds (*Helianthus annuus* L.). Water was provided in addition to these foods. Supplies were replenished at four day intervals.

### Mating behavior

Virgin female and male couples in four different combinations (unexposed couple; exposed female; exposed male; and exposed couple) were allowed to mate. All matings were digitally recorded (HDR-XR520V, Sony, Tokyo, Japan) for 13 h. After this mating period, the males were removed and all insects were kept in individual containers. Film analysis permitted evaluation of the latency to the first mating, the number of matings, the duration of each mating and the total mating duration for each couple. The number of couples that did not mate was always less than 2% and their results were not used in the statistical analysis.

### Reproduction and survival bioassays

Twenty to twenty-five mated couples per treatment were then separately monitored until death. The daily number of laid eggs/female, the number of egg masses/female, the number of eggs/egg-mass/female, egg hatching, the percentage of females laying eggs and the survival rates of each sex were recorded daily. Insects were recognized as dead when they were unable to walk after being prodded with a fine hair brush.

### Behavioral responses

Behavioral bioassays were conducted in arenas that were fully treated either with imidacloprid residues (at 0.042 μg a.i./cm^2^, the equivalent of 1% of the field rate dose), or with deionized water (control), following methods previously described elsewhere [48–52]. Briefly, the filter paper disks were impregnated with 1 mL of insecticide or water solution and, after drying for 20 min, the filter paper was placed in Petri dishes (135 × 20 mm). The inner walls of each Petri dish were coated with Teflon^®^ PTFE (DuPont, Wilmington, DE, USA) to prevent insects from escaping. The movement of each insect within the arena was recorded for 60 min using an automated video tracking system equipped with a CCD camera (ViewPoint Life Sciences Inc., Montreal, CA). The parameters that were recorded for each insect included the walked distance and the time spent walking. Twenty to twenty-five replicates, consisting each of a newly emerged (< 24 h old) virgin female or male, were used for each treatment. The insects used in these behavioral experiments faced the insecticide exposure for the first time as they were put at the arena. After each trial or replicate, the filter paper was replaced.

### Respiration rate bioassays

Respiration rates were evaluated using a CO_2_ Analyzer TR3 (Sable Systems International, Las Vegas, NV, USA), following previously described methods [50,53–55]. The average respiratory rate (CO_2_ production) was measured for 20 recently emerged female and male adults that were either unexposed or previously subjected to imidacloprid contact exposure for 60 min (at 0.042 μg a.i./cm^2^). The insects were placed in 25-mL chambers connected to a completely closed system. The chambers were connected to the system for 90 min before injecting CO_2_-free air into the chambers for 2 min at a rate of 600 ml/min. The air current directed the CO_2_ that was produced by insect respiration to an infrared reader connected to the system, allowing the immediate quantification of the CO_2_ that was produced. The insects were weighed before and after respiration rate determination on an analytical balance (Sartorius BP 210 D, Göttingen, Germany), and body mass variation was quantified.

### Testicle morphometry and histological structure

#### Testicle morphometry

Twenty newly emerged males (< 24 h old) were exposed for 48 h to dry imidacloprid residues (at 0.042 μg a.i./cm^2^) or to deionized water (control), as previously described. Five exposed and five unexposed males were used for the reproductive trait analysis just after the exposure and 10 days after the 48 h exposure period. Individual insects were dissected in phosphate buffer 0.1 M, pH 7.2, and the testicles were removed and photographed in a stereoscopic microscope ZEISS Stemi 2000-C with an attached digital camera. Using the Image- Pro Plus program (version 4.5 for Windows 98.), the length and width of the 10 testicles of dissected insects (five treated and five untreated) were measured. The same testes and accessory glands were used for the histological studies.

#### Testicle and accessory gland histological structure

For the histological analysis, the testes and accessory glands were fixed for 48 h in ethanol/acetic acid (3: 1) fixative solution, washed in 0.1 M phosphate buffer, pH 7.2, dehydrated in an ascending alcohol series of 30%, 50%, 70%, 90% (15 minutes each), and placed in two 100% alcohol baths (10 minutes each). Dehydration was followed by two baths of 4 h, each at room temperature. First these testes and accessory glands were placed in a mixture of historesin and ethanol (1: 1) and then in pure historesin. Successively, the testes and accessory glands were immersed in catalyzed historesin in silicone molds that were transferred to an oven at 58°C for 24 h. Histological sections (1.0 to 0.5 μm thick) were obtained using a Leica RM 2155 microtome with glass blades. They were transferred to histology slides, stained with Harris hematoxylin for 15 minutes, washed in tap water for 10 minutes, stained with eosin for 1 minute and rapidly rinsed with water. The histological sections were viewed and photographed using optical and light microscopy (Olympus BX-60) with an attached digital camera.

### Statistical analyses

The results of mating behavior (number of times and duration of mating), the time to the first viable egg, and first hatching were subjected to multivariate analysis of variance (MANOVA) to secure an overall error level of *P* < 0.05 with subsequent (univariate) analyses of variance of each trait, when appropriate (PROC GLM, SAS Institute, 2008). Walking behavior and respiration results were subjected to univariate analysis of variance (ANOVA) or a Kruskal-Wallis one-way ANOVA on ranks, when the assumptions of normality and homoscedasticity were not satisfied. The results of the survival bioassays were subjected to survival analysis, which was performed by using Kaplan-Meier estimators (Log-rank method) with SigmaPlot 12.0 (Systat Software, San Jose, CA, USA). When appropriate, regression analysis was performed using the curve-fitting procedure of SigmaPlot 12.0. Regression analyses were performed to detect trends in daily fecundity and fertility parameters that resulted in each treatment through time. The regression model was chosen based on parsimony, lower standard errors, and steep increases in R^2^ with model complexity. The regression models for each treatment were considered different from each other if the confidence limits of their parameters did not overlap. Data on testicles morphometry were submitted to univariate ANOVA and averages were tested by a *t* test at 0.05 probability. The assumptions of normality and homogeneity of variance were tested for in all parameters, and no data transformations were necessary (PROC UNIVARIATE, SAS Institute, 2008).

## Results

The multivariate analysis of variance (MANOVA) indicated significant overall effect of sublethal imidacloprid exposure for the group of traits encompassing the mating behavior (number of times and duration of mating) and latency for egg-laying and first hatching (*F*_*app*._ = 3.05; *df* = 12; *P* = 0.006).

### Mating behavior

**Fig 1:**
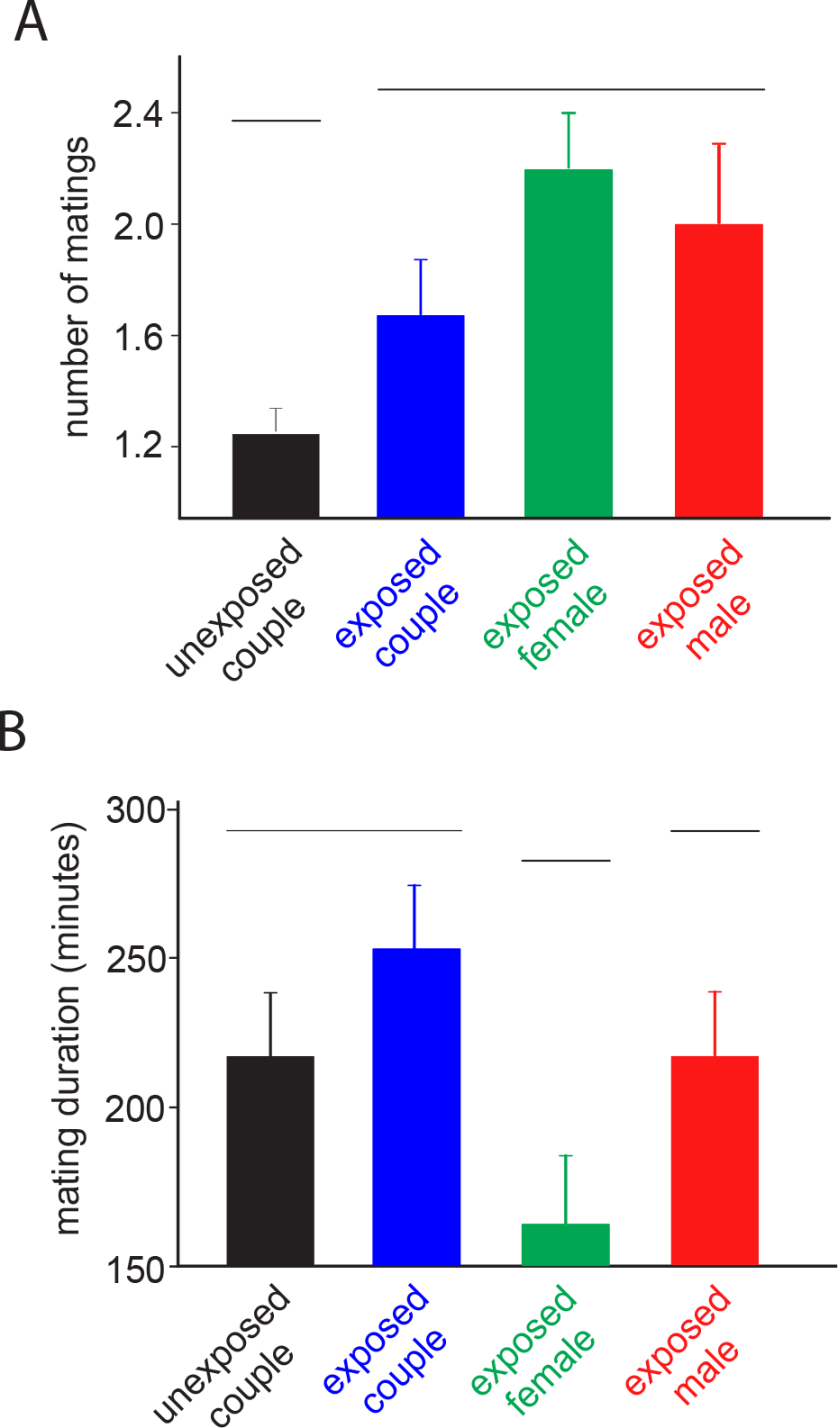
Imidacloprid-mediated effects on the mating behavior of *E*. *heros* couples. Number of matings (A) and average mating duration (B) for *E*. *heros* couples when both sexes were unexposed, both sexes were exposed, only females were exposed and only males were exposed. Couples of *E*. *heros* grouped by the same horizontal line did not differ according to a Tukey’s HSD test (*P* < 0.05).

Sublethal imidacloprid exposure did have significant effects on mating parameters (H = 8.79; *df* = 3; *P* = 0.032 and H = 9.02; *df* = 3; *P* = 0.029 for number of times and duration of mating, respectively). In fact, although all exposed couples mated more frequently than unexposed couples, couples in which only females were exposed mated for a shorter duration (Fig 1A and 1B).

### Survival and reproduction bioassays

Median survival time (LT_50_) ranged from 45.0 to 46.9 days for females and from 44.4 to 47.9 days for males. There were no significant differences among the four treatments for both sexes (χ^2^ = 0.24; *df* = 3; *P* = 0.97 and χ^2^ = 2,19; *df* = 3; *P* = 0.53 for females and males, respectively).

#### Fecundity and fertility responses

**Fig 2:**
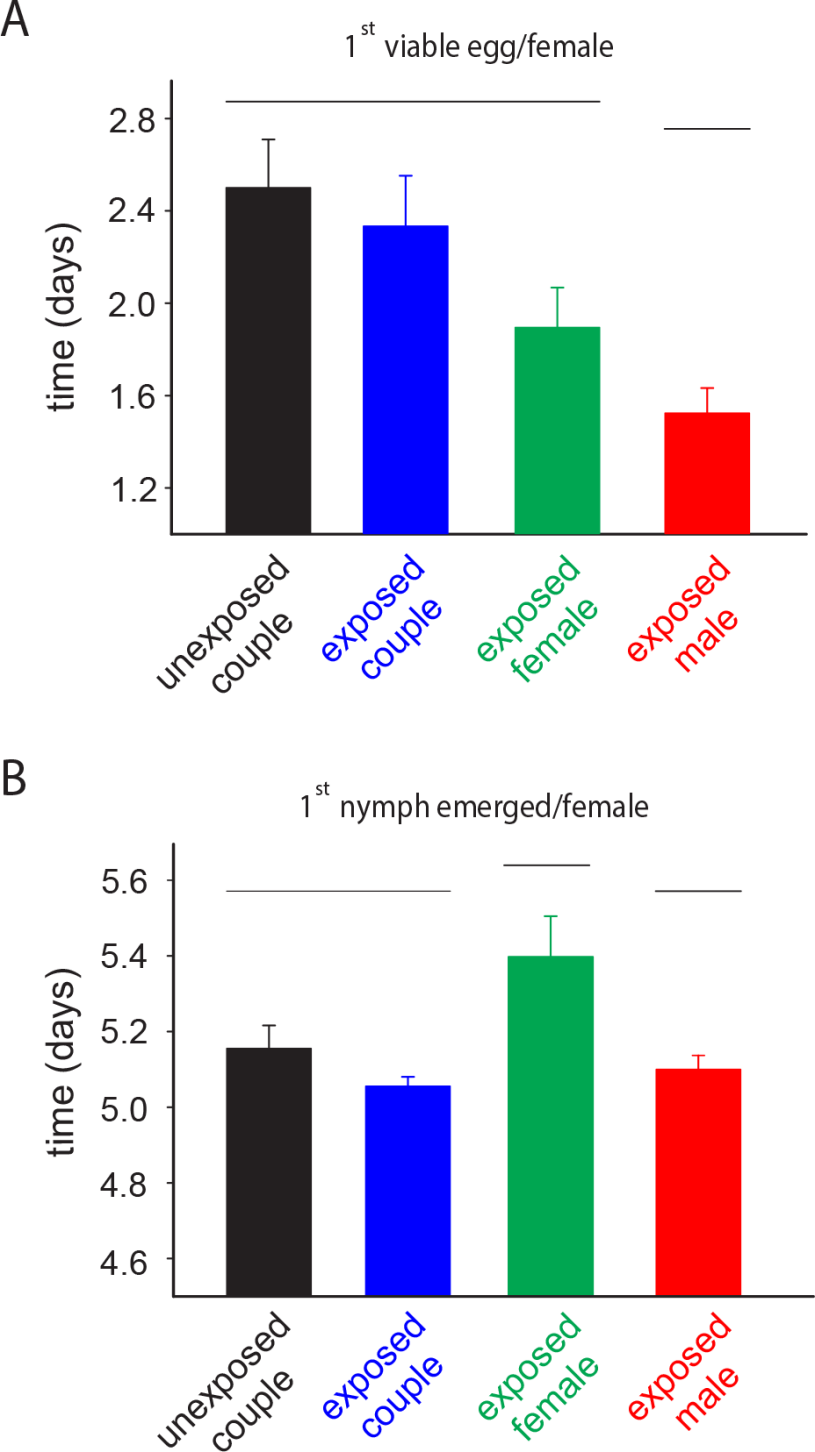
Imidacloprid-mediated effects on the reproductive outputs of *E*. *heros* couples. Effects of sublethal exposure to imidacloprid on the time to the first viable egg laid (A) and to the first nymph emerged (B). Couples of *E*. *heros* grouped by the same horizontal line did not differ according to a Tukey’s HSD test (*P* < 0.05)

Significant differences were found between the four treatments for the time to the first viable egg laid and to the first hatching (H = 10.75; *df* = 3; *P* = 0.013 and H = 9.68; *df* = 3; *P* = 0.021, respectively). While the time to the first viable egg laid was shorter for the couples where only the male was exposed (Fig 2A), the time to the first hatching was longer for couples where only the female was exposed (Fig 2B).

**Fig 3:**
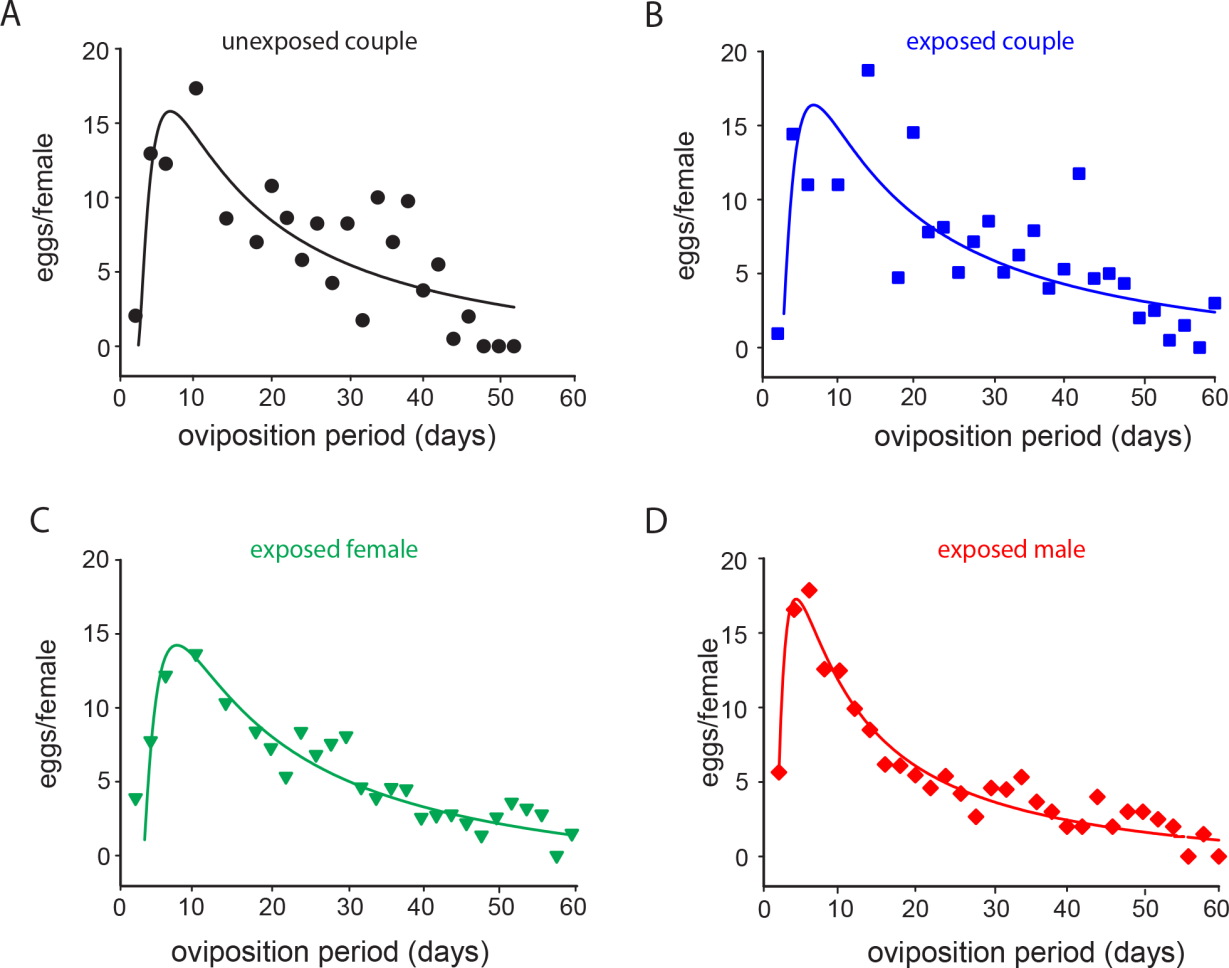
Imidacloprid-mediated effects on the daily fecundity of *E*. *heros* females. (A-D) Daily fecundity of *E*. *heros* females that were sublethally exposed to imidacloprid and to distilled water, coupled with insecticide-treated or insecticide-untreated partners. Lines represent the fit of daily fecundity results. Symbols represent means of the observed data.

**Fig 4:**
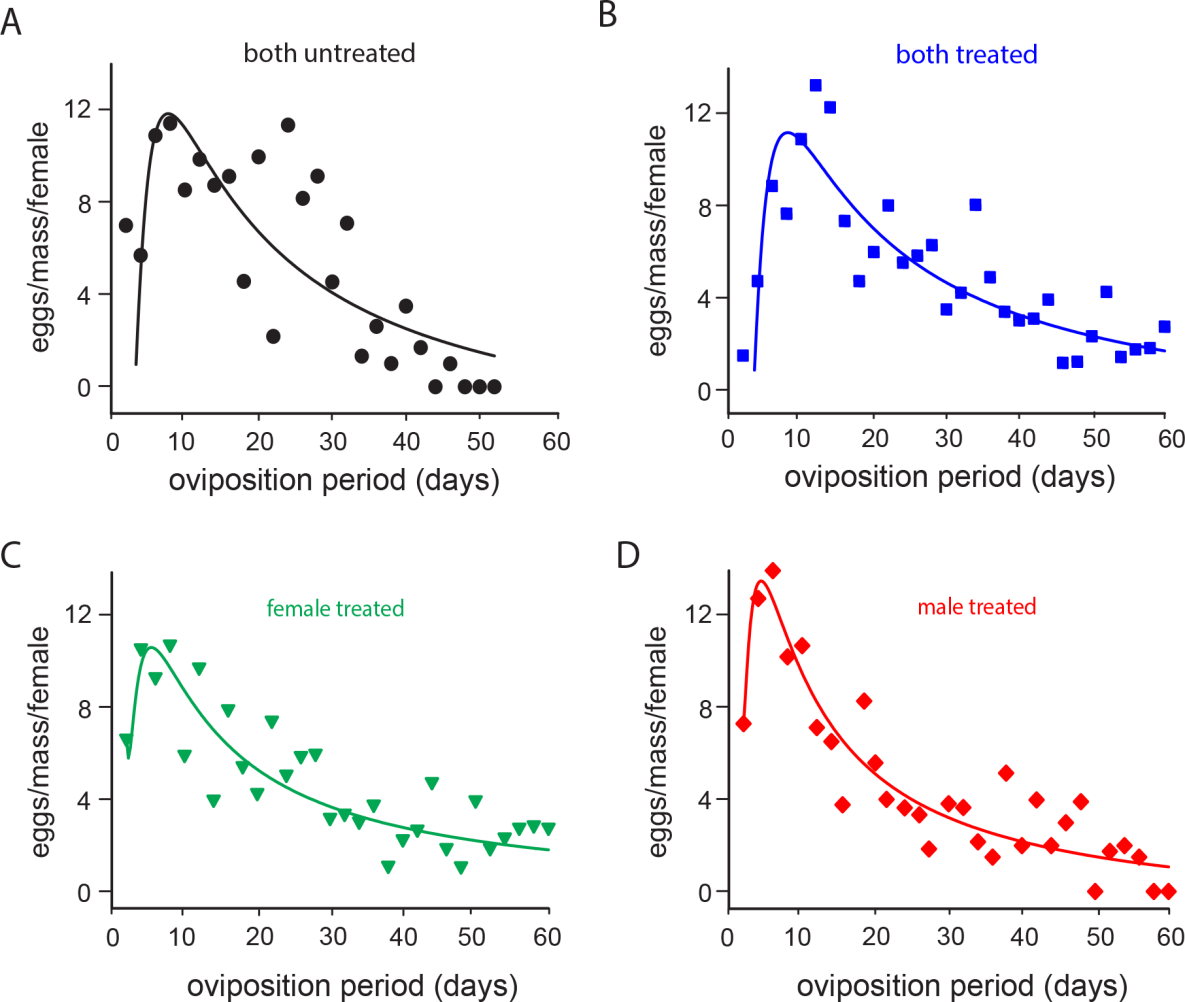
Imidacloprid-mediated effects on the egg-mass size. (A-D) Number of eggs/egg-mass of *E*. *heros* females that were sublethally exposed to imidacloprid and to distilled water, coupled with insecticide-treated or insecticide-untreated partners. Lines represent the fit of daily fecundity results. Symbols represent means of the observed data.

For all of the parameters of daily fecundity and fertility analysis, sublethal exposure to imidacloprid only affected the daily number of eggs laid (Fig 3, Table 1) and the number of eggs/egg masses (Fig 4, Table 1). Differences in the daily number of eggs were recorded between the two treatments when only females or males were exposed. Unexposed females mated with imidacloprid-exposed males exhibited higher daily fecundity rates in the beginning of the oviposition period compared to imidacloprid-exposed females mated with unexposed males (Fig 3). For the number of eggs/egg masses, differences were found for the three exposure combinations where either both or at least one insect was exposed (Fig 4). The highest number of eggs per mass was recorded during the first two weeks of the oviposition period for couples when only the male was exposed to imidacloprid (Fig 4). Despite the fact that the results obtained for the percentage of egg hatched and number of eggs/egg-masses fit well with exponential models, no significant differences were observed among treatments (Table 1).

**Table 1:** Summary of non-linear regression analyses of fecundity parameters (shown in Figs 3 and 4).

Variable	Model	Treatment	Estimated parameters* (±SE)				df_error_	F	*P*	*R*^2^
			*a*	*b*	*c*	*y*_*0 or*_ *x*_*0*_				
Number of eggs/female (Fig 2)	y = y_0_+(a/x)+(b/x^2^)+(c/x^3^)	Both untreated	132.9 (65.0–200.9)ab	-294.6 (-497.2–92.0)	165.8 (24.6–307.1)	-2.1 (-6.1–2.0)	22	11.6	0.0002	0.66
		Both treated	139.2 (84.4–193.9)ab	-314.2 (-485.6–142.8)	178.0 (55.9–300.2)	-1.9 (-4.7–0.91)	32	17.2	<0.0001	0.64
		Only female treated	146.1 (127.5–164. 6)a	-364.2 (-423.0–305.4)	225.2 (183.0–267.4)	-3.1 (-4.0–2.1)	36	122.4	<0.0001	0.91
		Only male treated	91.6 (75.3–107.9)a	-130.1 (-183.2–76.9)	45.9 (7.2–84.6)	-1.8 (-2.7–0.9)	34	196.1	<0.0001	0.94
Number of Eggs/mass (Fig 3)	y = y_0_+(a/x)+(b/x^2^)+(c/x^3^)	Both untreated	73.6 (10.3–137.0)ab	-147.6 (-335.6–40.5)	74.4 (-57.2–206.0)	1.4 (-3.1–5.9)	20	5.9	0.0057	0.51
		Both treated	120.6 (94.8–146.4)b	-321.2 (-405.1–237.2)	204.1 (143.1–265.1)	-2.1 (-3.5–0.7)	34	38.6	<0.0001	0.79
		Only female treated	69.9 (48.3–91.6)a	-134.9 (-205.9–63.9)	72.0 (20.2–123.8)	-0.4 (-1.5–0.8)	36	40.9	<0.0001	0.79
		Only male treated	75.8 (57.6–94.1)a	-117.8 (-175.9–59.7)	50.6 (8.7–92.6)	-1.3 (-2.4–0.3)	30	103.7	<0.0001	0.92
Proportion of egg hatching	y = a/(1+exp (-(x-x_0_)/b))	Both untreated	0.7 (0.5–0.8)a	-3.2 (-5.8–0.6)	-	16.1 (13.1–19.1)	23	27.5	<0.0001	0.72
		Both treated	0.5 (0.3–0.7)a	-3.8 (-7.3–0.2)	-	13.9 (9.2–18.7)	24	22.1	<0.0001	0.67
		Only female treated	0.7 (0.4–0.9)a	-7.5 (-12.5–2.5)	-	17.3 (8.7–25.9)	37	45.2	<0.0001	0.72
		Only male treated	0.8 (0.,3–1.4)a	-7.1 (-12.2–2.0)	-	11.7(0.4–2.9)	32	45.8	<0.0001	0.75
Number of egg-masses/females	y = a*exp (-0,5*((x-x_0_)/b)^2^)	Both untreated	1.4 (1.0–1.7)a	9.1 (4.2–14.1)	-	8.8 (5.0–12.5)	22	5.4	0.0135	0.35
		Both treated	1.5 (1.1–1.8)a	12.0 (6.4–17.7)	-	12.5 (8.0–17.0)	32	5.4	0.0099	0.27
		Only female treated	1.4 (1.1–1.6)a	16.8 (8.9–24.8)	-	13,0 (7.1–18.9)	36	5.9	0.006	0.26
		Only male treated	1.4 (1.2–1.6)a	10.9 (6.8–15.0)	-	5.1 (0.2–10.1)	31	39.9	<0.0001	0.73

*Parameter values followed by different letters in the columns were significantly different (based on non-overlapping of confidence limits).

### Behavioral responses

**Fig 5:**
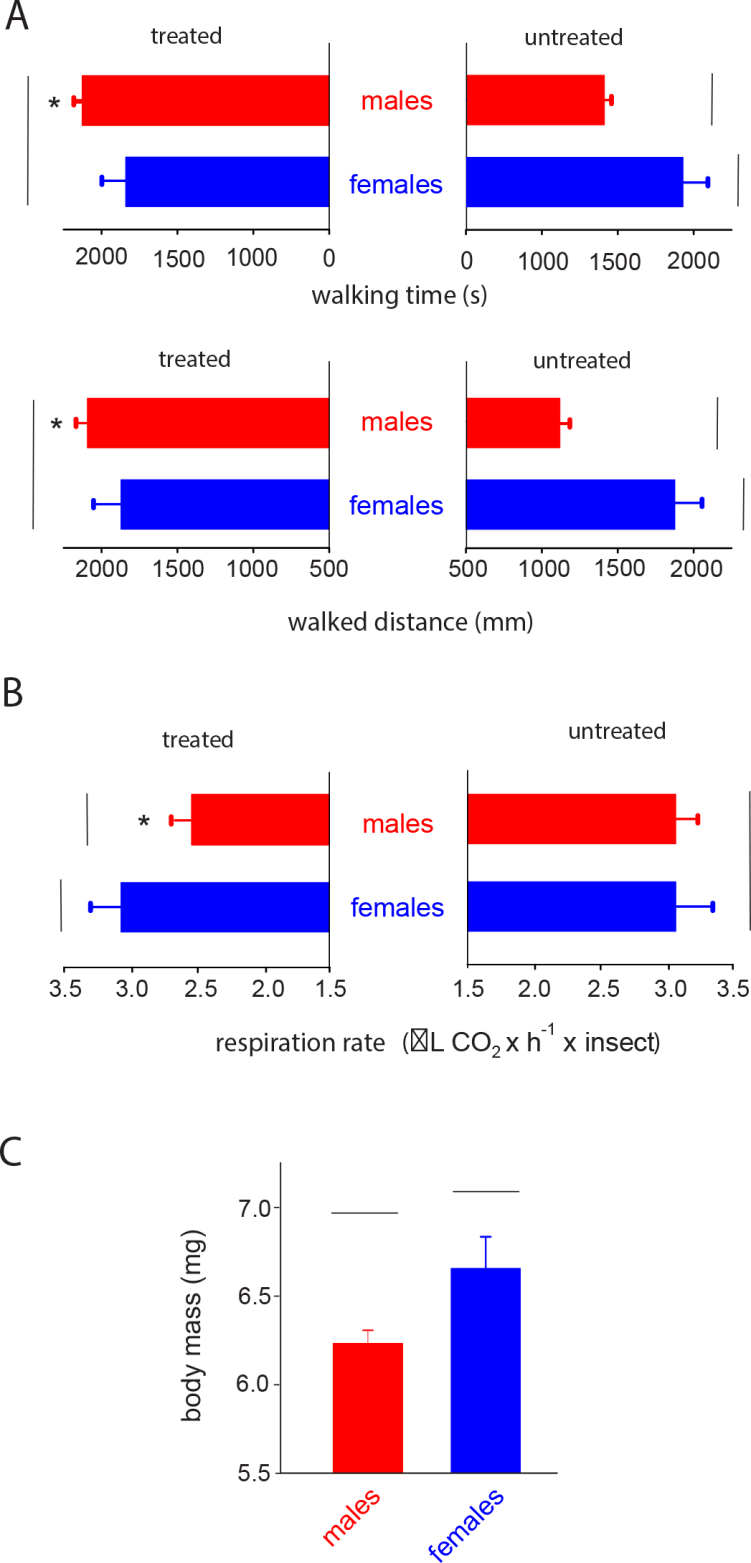
Behavioral and physiological responses of E. heros after being sublethaly exposed to imidacloprid. Walking activities (A) and respiration rates (B) of recently emerged (< 24 h) *E*. *heros* males and females sublethally exposed to imidacloprid. (C) Body masses of recently emerged *E*. *heros* males and females. Each histogram represents the mean (± SE) of 20 replicates. Vertical lines (A and B) and horizontal lines (C) indicate no differences according to a Tukey´ s HSD test (*P* < 0.05). Asterisks indicate significant differences between responses of imidacloprid-treated and untreated insects.

As far as their locomotory performance in uncontaminated arenas, females significantly differed from males in both the distance walked (*H* = 6.14; *df* = 1; *P* = 0.013) and the time spent walking (*H* = 6.39; *df* = 1; *P* = 0.011), with higher values recorded for untreated females (Fig 5). Sublethal exposure to imidacloprid in treated arenas significantly affected the distance walked (*H* = 7.42; *df* = 1; *P* = 0.006) and time spent walking (*H* = 8.58; *df* = 1; *P* = 0.003) of males compared to uncontaminated arenas. In contrast, no differences were observed for females (*Walked distance*: *H* = 0.017; *df* = 1; *P* = 0.88. *Walking time*: *H* = 0.0035; *df* = 1; *P* = 0.95) between treated and untreated arenas (Fig 5).

### Respiration rates

The respiration rate was significantly reduced for imidacloprid-exposed males compared to unexposed males (*F* = 6.32; *P* = 0.02). On the other hand, no significant differences were observed between exposed and unexposed females (*F* = 1.65; *P* = 0,21) (Fig 5B). Body mass variation (before and after determination of respiration rates) was found to be similar for both exposed and unexposed insects, although, in general, females showed statistically (*H* = 8.60; *df* = 1; *P* = 0.0033) higher mass compared to males (Fig 5C).

### Testicle structure and morphometry

**Fig 6:**
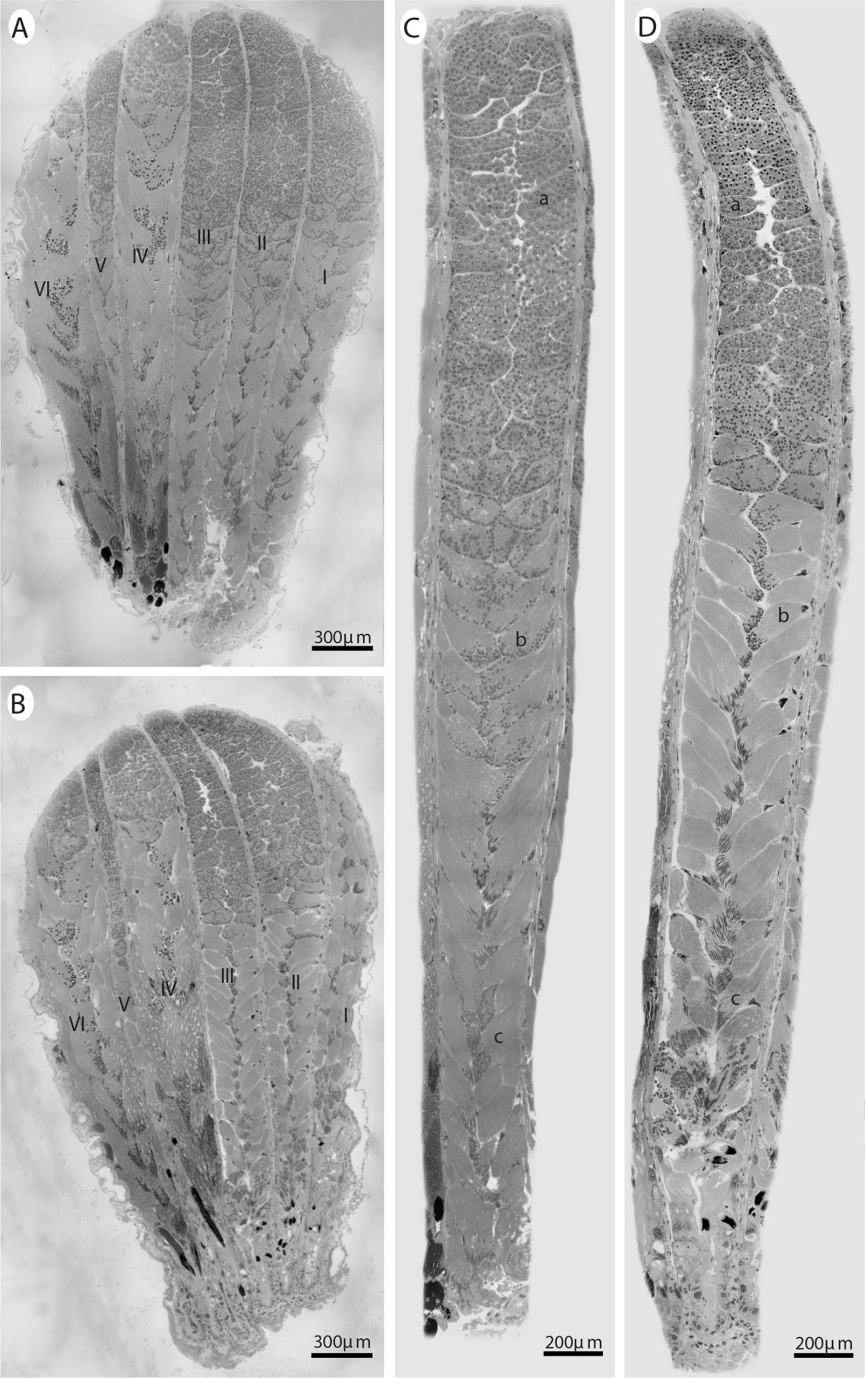
Histological sections of *E*. *heros* testicles stained with hematoxylin and eosin. Longitudinal sections of the testicles and testicular follicles of 10 day old treated (A and C) and untreated (B and D) males of *E*. *heros*. In the 3^rd^ follicle magnification (C and D), cysts can be observed with spermatids at early (***a***), intermediate (***b***) and more advanced (***c***) stages of maturation.

Structural analysis of reproductive traits in *E*. *heros* showed that the testicles are elongated globes of red yellowish color formed by each of 6 follicles filled by grouped germ cell cysts, with the diameter of the 5^th^ follicle narrower than those of the other follicles (Fig 6). The average length and width of the testes were 3.5 ± 0.13 and 1.86 ± 0.12 mm for unexposed males and 3.61 ± 0.23 and 1.92 ± 0.21 mm for exposed males. Spermatogenesis began in the larger portion of the testicles and ended in the narrowest part of the testicles. No visually detectable differences were found between exposed and unexposed males for cysts. This was the case for both early and advanced stages of spermatid maturation. The size of the 4^th^ and 6^th^ follicles was larger than other follicles in both imidacloprid exposed and unexposed males (Fig 6).

No significant differences (*F* = 0.67; *P* = 0.42 for the testicle width and *F* = 0.70; *P* = 0.41 for the testicle length) were found between sexually mature males exposed or not exposed at 10 days after sublethal imidacloprid exposure. However, just following exposure, treated males had significantly (*F* = 8.21; *P* = 0.010) wider testicles (*Untreated males*: 1.54 ± 0.036 mm and *Treated males* 1.68 ± 0.034 mm).

**Fig 7:**
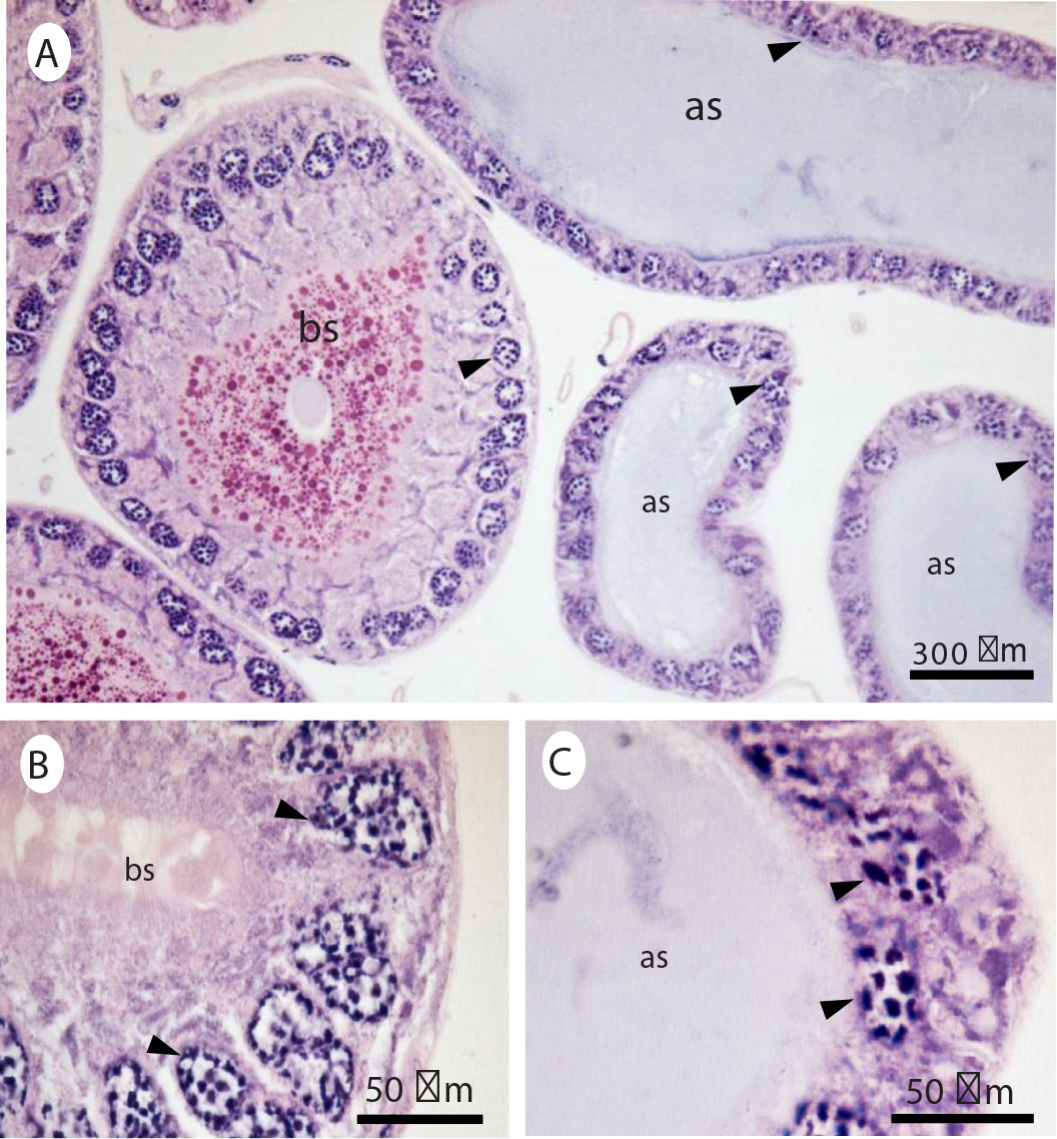
Histological sections of *E*. *heros* accessory glands stained with hematoxylin and eosin. Sections of the accessory glands of 10 day old males of *E*. *heros*, stained with hematoxylin and showing basic (***bs***) and acidic (***as***) secretions Black arrows indicate the cell nucleus.

No differences were detected in accessory glands between imidacloprid-exposed and unexposed males for the two evaluation dates (Fig 7). Nevertheless, different colors, resulting from staining with hematoxylin, indicated the existence of both acidic and basic contents in the accessory glands. However, this difference was not associated with imidacloprid exposure, as both exposed and unexposed insects displayed the two types of reactions.

## Discussion

In the present work, we evaluated whether insecticide-mediated stress in early adulthood would stimulate hormetic responses related to the sexual success of *E*. *heros* males. Although exposure to imidacloprid did not impact the survival abilities of *E*. *heros* males, these insects were capable of recognizing and avoiding the impending stress posed by imidacloprid-treated areas. When sublethally stressed by this insecticide, these males increased their mating frequency and induced higher fecundity rates in unexposed females of *E*. *heros*.

There is increasing evidence that environmental stressors, such as insecticides, can have stimulatory or beneficial effects at low exposure levels, despite being toxic at higher levels [3,4,10,11,56]. This phenomenon has been referred as insecticide-induced hormesis. Despite the fact that it has been identified in a number of arthropod species [15,16,18,44,57–59], the vast majority of such investigations have focused on the reproductive performance of adult females. These studies have reported that insect females sublethally exposed to insecticides exhibit compensatory effects, resulting in a higher reproductive performance and a shorter life span [18,44,60,61].

Insecticide-mediated alterations on the reproductive performance of insect males are frequently neglected. Here, sublethal exposure to imidacloprid increased the sexual success of *E*. *heros* males by increasing their mating frequency, which resulted in higher reproductive outputs of their unexposed female partners. Males are known to typically increase their fitness by increasing their mating frequency, whereas females often suffer reduced fitness from multiple matings, potentially leading to sexual conflict over mating [21–24]. Such gains in male fitness success may favor the evolution of male traits that entice females to mate multiply, thereby increasing the probability that males will sire offspring [21–25]. However, additional study is required to evaluate precisely what property of sublethal exposure to imidacloprid is responsible for the responses of males. Furthermore, during the mating period, insect males may use a variety of mechanisms (e.g., sperm mobility, sperm storage, stimulation of ovulation/oviposition, and egg protection) to improve their chances of transferring genetic material [62–65]. Sublethally stressed *E*. *heros* males could also alter some of these mechanisms and/or manipulate their accessory gland secretions to increase their fitness, as has already been shown in other insect species [15,66,67].

Our results on the sexual success of *E*. *heros* females sublethally exposed to imidacloprid were somehow contradictory. Imidacloprid-exposed females possibly attempted to increase their reproductive performance by mating multiply with unexposed males, which may potentially compensate for the shortened duration of mating events recorded for these couples. Although controversial, by increasing their mating frequency, multiply mated females may increase their fitness by receiving more male-contributed materials [21,22,24]. However, these multiply mated females may also suffer reduced fitness because multiple matings have costs, such as increased time and energy expenditures, increased risk of predation and increased risk of being intoxicated by undesirable male materials [21,22,24]. For instance, we recorded a significant delay in the time needed for the first emerged nymph of couples when only the female was sublethally exposed to imidacloprid, which may reflect the potential costs of these multiple matings.

While both males and females of *E*. *heros* were able to avoid imidacloprid-contaminated areas, males exhibit rather different walking and respiratory responses than females when they were forced to face the stress of imidacloprid. The higher walking activity of sublethally exposed males may reflect a higher capacity to detect the presence of imidacloprid molecules. Although not addressed in detail here, differential physiology, morphology and distribution of sensilla have been recorded in males and females of some insect species [68,69]. In addition, *E*. *heros* males and females may be equipped with different subtypes of nAChRs. Imidacloprid has distinct pharmacological profiles in diverse subtypes of insect nAChRs [34–38]. Furthermore, such alterations in walking behavior has been shown to be a strategy used by insects to overcome the actions of natural and synthetic insecticides [19,29,48–50,52]. Such higher walking activity in exposed males of *E*. *heros* may have contributed not only in fleeing from insecticide-contaminated areas (mitigating the threats of insecticide stress) but also in enhancing their mating success due to an increase in the rate of encounter with mating partners [26,27,70]. However, greater locomotory ability may also result in additional encounters with predators [26,27,70].

Intriguingly, despite these imidacloprid-induced increases in the locomotory activities of *E*. *heros* males, our respirometry results showed a clear decrease in the respiration rates of these insects. Generally, higher levels of walking activity would be expected to result in higher metabolism and, consequently, a higher respiration rate. However, reductions in respiratory responses have been reported in arthropods that were sublethally exposed to pesticides [31,71–73] as a result of the impairment of oxidative phosphorylation processes [31–33].

Thus, our results showed that *E*. *heros* males, when coupled with unexposed females, hormetically increase their sexual success when facing potential survival threats, such as sublethal exposure to imidacloprid. Taking into consideration that the morphometric experiments performed in this study were not able to capture any significant difference between reproductive tracts of exposed and unexposed *E*. *heros* males, future experiments that aim to evaluate potential alterations in the abundance and types of spermatozoids or to determine and quantify the contents of the accessory glands are sorely needed, as studies of this type will likely provide novel insights into these processes. Furthermore, additional experiments with individuals sublethally exposed to other insecticides concentrations (e.g., 3% and 10% of field rate for imidacloprid) are likely to further clarify the potential differences that may result from sublethal exposure in *E*. *heros*.

## Supporting Information

S1 Data Raw data used in all the statistical analysis. (XLSX)
